# Fracture Resistance of Direct versus Indirect Restorations on Posterior Teeth: A Systematic Review and Meta-Analysis

**DOI:** 10.3390/bioengineering11060536

**Published:** 2024-05-24

**Authors:** Carol Moussa, Guillaume Savard, Gael Rochefort, Matthieu Renaud, Frédéric Denis, Maha H. Daou

**Affiliations:** 1 Faculty of Dentistry, University of Tours, 37032 Tours, France; guillaume.savard@univ-tours.fr (G.S.); gael.rochefort@univ-tours.fr (G.R.); matthieu.renaud@univ-tours.fr (M.R.); frederic.denis@univ-tours.fr (F.D.); maha.daou@univ-tours.fr (M.H.D.); 2 Department of Restorative Dentistry, Faculty of Dental Medicine, Saint-Joseph University, Beirut 1107 2180, Lebanon; 3 Division of Education, Ethics, Health, Faculty of Medicine, University of Tours, 37044 Tours, France; 4 Department of Medicine and Bucco-Dental Surgery, Tours University Hospital, 37044 Tours, France; 5 INSERM, Imaging Brain & Neuropsychiatry iBraiN U1253, 37032 Tours, France; 6 N2C Laboratory, UMR INSERM U 1069, University of Tours, 37032 Tours, France; 7 Department of Pediatric Dentistry, Faculty of Dental Medicine, Saint Joseph University, Beirut 1107 2180, Lebanon; 8 Division of Biomaterials, Craniofacial Research Laboratory, Saint Joseph University, Beirut 1107 2180, Lebanon

**Keywords:** fracture resistance, direct restoration, indirect restoration, posterior teeth

## Abstract

The aim of this systematic review and meta-analysis was to compare static compression forces between direct composite resin restorations and indirect restorations for posterior teeth. All studies comparing mechanical properties of direct versus indirect restorations of posterior teeth were included from 2007 up to February 2024. A meta-analysis was conducted for static compression fracture resistance. Medline, Central, and Embase databases were screened. Twenty-four articles were included in the qualitative synthesis, and sixteen studies were finally included in the quantitative synthesis. There was no difference in terms of fracture resistance between direct and indirect restorations for posterior teeth (*p =* 0.16 for direct and indirect composite resin restorations and *p =* 0.87 for direct composite resin restorations and indirect ceramic restorations). Also, sub-group analysis with or without cusp coverage in each group revealed no discernable difference. Based on this study, it can be concluded that the choice between direct and indirect restoration approaches may not significantly impact fracture resistance outcomes. There was no statically significant difference between direct and indirect restorations for posterior teeth in all cases of restorations with or without cusp coverage and no matter the used materials. However, to better evaluate these materials, further studies are warranted.

## 1. Introduction

Throughout the evolution of dentistry, the achievement of durable restorations for posterior teeth has been a constant objective. These restorations play a pivotal role in maintaining oral health and overall well-being since they are essential for efficient mastication, proper occlusal function, and maintaining the stability of the dental arch, while contributing to the structural integrity of the dentition. From ancient civilizations using materials like gold and silver amalgam [1] to modern advancements in composite resins and ceramic restorations [2,3,4,5], the quest for optimal solutions continues.

Today, dental practitioners have the possibility to choose between various techniques for restoring posterior teeth [6,7,8,9]. Restorations can be either directly prepared on the tooth structure, or indirectly fabricated and then bonded to the tooth; each technique should be considered with its own advantages and challenges [10,11,12,13].

However, among the vast array of restorative materials and techniques, there is a notable absence of comprehensive evidence regarding the mechanical characteristics of posterior teeth restorations through direct and indirect methods. Previous systematic reviews have compared clinical performance of direct versus indirect restorations; however, these systematic reviews did not focus on the mechanical properties of each restoration [14,15,16,17,18,19,20,21].

In this context, it is important to evaluate the impact of direct versus indirect restorations on the mechanical performance of tooth structures by rigorously analyzing studies that directly compare these restoration techniques in terms of fracture resistance, specifically in terms of static compression strength.

The aim of this study was to evaluate the existing literature and compare static compression forces between direct composite resin restorations and indirect restorations for posterior teeth in order to provide evidence-based insights to inform clinical decision-making and guide future research in this area. This study focused on inlay and onlay restorations, excluding crown/complete coverage restorations.

## 2. Materials and Methods

### 2.1. Search Strategy and Study Selection

All studies comparing mechanical properties of direct versus indirect restorations of posterior teeth were considered for inclusion.

A systematic literature review was conducted following the Cochrane Guidelines and Preferred Reporting Items for Systematic Reviews and Meta-Analyses (PRISMA), up to February 2024. The search included the bibliographic databases of Medline, Embase, and Central. Only articles published in English were selected.

A review protocol was developed prior to the study. The PICO model employed for this study was outlined as follows: Is there a difference in mechanical properties (fracture resistance) (O) of direct (C) vs. indirect restorations (I) for the restoration of posterior teeth (P)? Identification and selection of the studies were carried out in accordance with PRISMA criteria [22]. Separate searches were performed using search strings (Table 1).

### 2.2. Selection Criteria

Two of the authors (MD and CM) conducted the article selection process. Only original studies that compared direct vs. indirect restorations of posterior teeth were eligible for inclusion. The systematic review included only in vitro and finite element analysis (FEA). Subsequently, only in vitro studies investigating compressive forces were analyzed in the meta-analysis. Initially, the titles and abstracts of the articles were screened to determine their adherence to the inclusion criteria. Articles passing the initial screening underwent a full-text analysis for final inclusion confirmation. Studies lacking primary data (letters to the editor/authors, case reports and commentaries) and conference abstracts were excluded from consideration. Additionally, the references of selected studies were manually reviewed to identify any additional relevant studies.

### 2.3. Data Extraction and Analysis

All studies meeting the inclusion criteria were identified and reviewed. Disagreement was resolved by consensus.

Data were extracted from each selected study. The extracted data encompass fracture resistance test values measured in Newtons, specifically conducted utilizing universal testing machines for static compressive forces. By narrowing the scope to compressive force assessments, the dataset ensures consistency and comparability across studies, facilitating robust quantitative analysis. These meticulous selection criteria underscore the rigor and precision applied in the extraction process, fostering confidence in the reliability and relevance of the synthesized findings for further investigation and interpretation within the domain of material fracture resistance.

For the meta-analysis, a software program, Review manager v5.4.1 (Cochrane Collaboration, Oxford, UK), was used to estimate the odds ratio (OR) with 95% confidence intervals (CIs). As only means and standard deviations (SDs) are permitted for the computational aspect of meta-analyses, a validated mathematical model was utilized to convert median (range) values to mean (SD) for studies that reported medians and ranges. Continuous data were analyzed using the Mantel–Haenszel Chi^2^ test and expressed as the weighted mean difference (WMD) with 95% confidence interval (CI), while dichotomous data were analyzed using the inverse variance method and expressed as odds ratios (ORs) or relative risks (RRs) with a 95% CI. A *p* value < 0.05 was considered significant. Heterogeneity was assessed using a Chi^2^ test on N−1 degrees of freedom, with statistical significance set at an alpha risk of 0.05 and utilizing the I^2^ test [23].

Pooled estimates were calculated using the random effect model to accommodate study heterogeneity. Potential publication bias was evaluated through funnel plot analysis for each outcome. All statistical analyses were conducted using Review manager v5.4.1 (Cochrane Collaboration).

### 2.4. Selection Criteria

**Figure 1:**
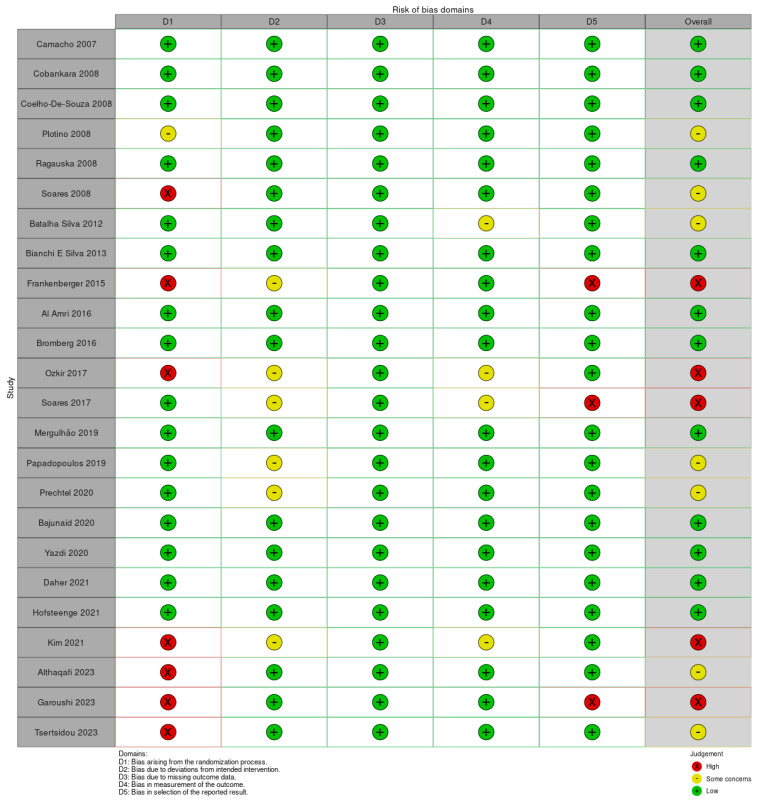
Risk of bias RoB2 assessment for the studies [24,25,26,27,28,29,30,31,32,33,34,35,36,37,38,39,40,41,42,43,44,45,46,47].

Studies were assessed for a risk of bias using the Cochrane risk of bias tool (ROB2 tool) (Figure 1). Performance bias was deemed low if a researcher other than the clinician was responsible for the randomization. If the clinician was responsible for this, it was scored as “some concerns”. In cases where no randomization procedure was outlined, it was categorized as “high risk”. Detection bias was considered “low risk” if a researcher other than the clinician assessed the restoration; otherwise, detection bias was deemed “high risk”.

## 3. Results

**Figure 2:**
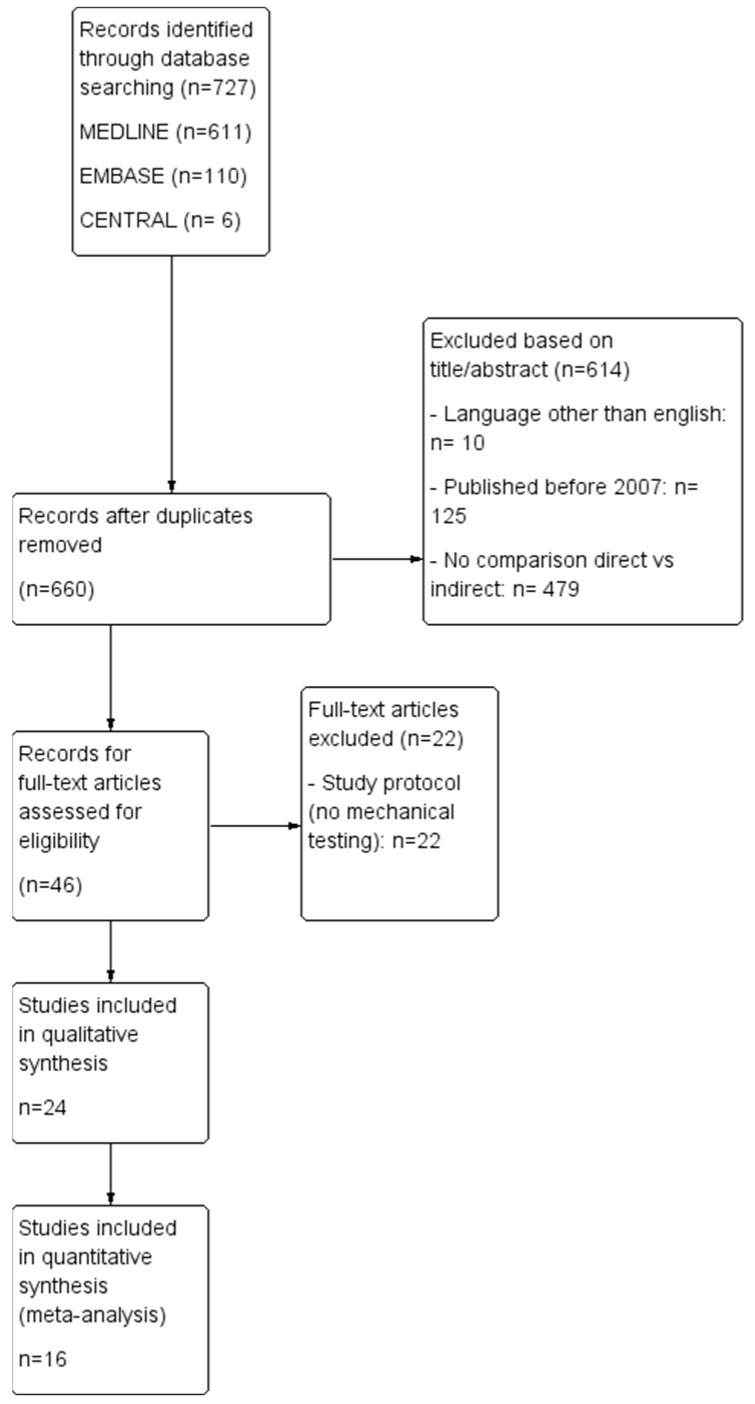
Study flow diagram of inclusion process for the literature concerning mechanical properties of direct vs. indirect restorations of posterior teeth.

A total of 727 potential publications were initially identified, of which 614 articles were excluded after abstract screening (due to language restriction, date of publication, irrelevant data). Forty-six full-text articles underwent eligibility assessment, and only twenty-four were included in the qualitative synthesis. Subsequently, sixteen studies met the criteria for quantitative synthesis (Figure 2).

Characteristics of included studies comparing direct vs. indirect restorations are presented in Table 2. A total of twenty-four studies met the inclusion criteria. The extracted data from the selected studies encompasses a comprehensive array of key parameters essential for analysis. Beyond basic bibliographic details such as year of publication, authors, and study title, the dataset included pivotal information regarding study type, delineating between in vitro and finite element analysis (FEA) studies. Furthermore, it specifies the study population by elucidating the number and kind of teeth included and denotes whether endodontic treatment was conducted. Crucially, the cavity configuration is meticulously detailed, with precision on whether cusp reduction was implemented or not. Each group’s composition is outlined through the number of specimens, while the evaluated direct and indirect materials are clearly specified. Additionally, the dataset indicates if aging processes like thermocycling were undertaken. Then the type of mechanical testing employed is specified to determine when static compression forces were used. Finally, it discerns whether an evaluation of fracture patterns was conducted. The evaluation of fracture pattern holds paramount importance in clinical dentistry as it provides crucial insights into the extent and nature of structural damage incurred by dental restorations under compressive forces. By categorizing fracture patterns, clinicians can assess the severity of damage and determine the feasibility of restoration options. This evaluation aids in decision-making regarding treatment modalities such as endodontic therapy or tooth extraction. Moreover, understanding the distribution and morphology of fractures guides clinicians in selecting appropriate restorative materials and techniques to optimize long-term outcomes for patients. While descriptive in nature, the evaluation of fracture pattern complements quantitative assessments of fracture resistance, collectively enhancing our understanding of the clinical performance of dental materials and restorative approaches.

The comparison between direct and indirect restorations was meticulously structured into two distinct groups to facilitate comprehensive analysis. The first group compares direct composite resin with indirect composite resin, while the second group compares direct composite resin with indirect ceramic restorations. Each of these primary groups was further subdivided into two distinct sub-groups: one with no cusp reduction and the other with cusp reduction. This deliberate subdivision was implemented to ensure comparability and enable a nuanced exploration of the impact of cusp coverage on restoration performance across different material types. By organizing the comparison in this manner, the dataset accounts for key variables and enhances the reliability and validity of the ensuing analysis.

### 3.1. Direct vs. Indirect Composite Resin Restorations

**Figure 3:**
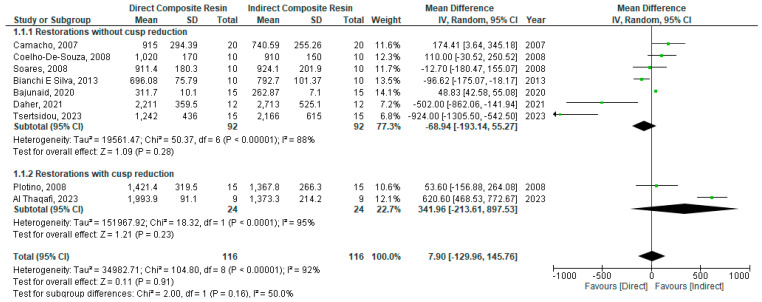
Forest plot of comparison of fracture resistance of direct vs. indirect composite restorations for the restoration of endodontically treated posterior teeth [24,26,27,29,31,40,42,45,47].

Regarding the comparison between direct and indirect composite resin restorations, a random model was used for analysis as there was a high degree of heterogeneity (I^2^ = 92%). There was no statistically significant difference in fracture resistance between direct and indirect restorations when composite is used for the restoration of posterior teeth (WMD: −7.09 [95%CI: −129.96, 145.76]; *p =* 0.16) (Figure 3).

#### Sub-Group Analysis: With vs. without Cusp Reduction

A sub-group analysis restorations with/without cusp reduction was performed using a random model as there was a high degree of heterogeneity (I^2^ = 95% and 88%, respectively). There was no statistically significant difference in fracture resistance between direct and indirect restorations when composite is used for the restoration of posterior teeth whether with or without cusp reduction: (WMD: 341.96 [95%CI: −213.61, 897.53]; *p =* 0.23) and (WMD: −68.94 [95%CI: −193.14, 55.27]; *p =* 0.28), respectively (Figure 3).

### 3.2. Direct Composite Resin vs. Indirect Ceramic Restorations

**Figure 4:**
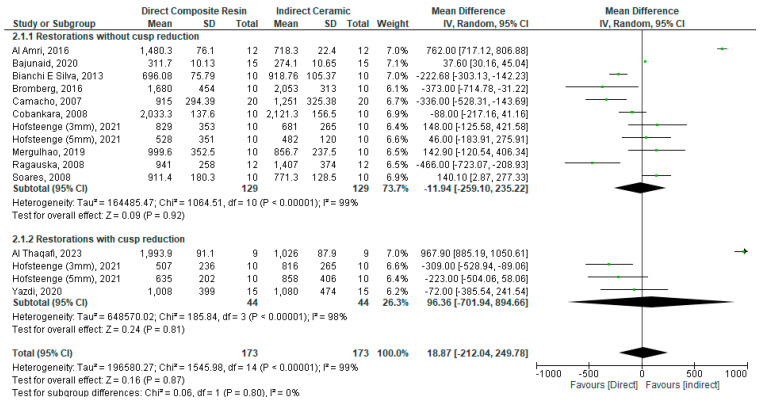
Forest plot for the fracture resistance of direct composite vs. indirect ceramic restoration of endodontically treated posterior teeth [24,25,28,29,31,33,34,37,40,41,43,45].

Another analysis was conducted for direct composite vs. indirect ceramic restoration of posterior teeth using a random model due to a high degree of heterogeneity (I^2^ = 99%). There was no statistically significant difference in fracture resistance between direct composite and indirect ceramic restorations (WMD: 18.87 [−212.04, 249.78]; *p =* 0.87) (Figure 4).

#### Sub-Group Analysis: With vs. without Cusp Reduction

A sub-group analysis restorations with/without cusp reduction was performed using a random model, as there was a high degree of heterogeneity (I^2^ = 98% and 99%, respectively). There was no statistically significant difference in fracture resistance between direct and indirect restorations when ceramic is used for the restoration whether with or without cusp reduction: (WMD: 96.36 [−701.94, 894.66]; *p =* 0.81) and (WMD: −11.94 [−259.10, 235.22]; *p =* 0.92), respectively (Figure 4).

## 4. Discussion

Dental restorations are subjected to various mechanical stresses during mastication, which can lead to failure if the material’s fracture resistance is inadequate. As mentioned earlier, dental restorations for posterior teeth can be prepared either directly or indirectly, each method possessing distinct characteristics. Even when focusing solely on mechanical attributes, this consideration encompasses multiple variables.

On one hand, direct composite restorations are a faster and less expensive choice [48]. However, as stated by Kaisarly and El Gezawi in their literature review, the polymerization shrinkage inherent to composite resins during curing can lead to microleakage, marginal gaps, and reduced fracture resistance [49]. Soares et al. recognized that shrinkage stress and its clinical implications are determined by many factors, highlighting the complexity of this topic beyond initial assumptions [50].

On the other hand, indirect restorations can grant improved marginal adaptation, as concluded by Lima et al. and by Santos and Busato [51,52]. Indirect restorations enable reduced polymerization shrinkage and more anatomy [53] control while allowing for a variety of material choices going from indirect composite resins to different kinds of ceramic restorations [54].

Fracture resistance of dental restorations is a critical parameter, as it directly influences the longevity and durability of the restoration, as well as the overall health of the tooth [9].

Batalha-Silvaa et al. [30] and Soares et al. [36] both used cyclic-load-to-failure tests in their comparison. In the first study [30], CAD/CAM MZ100 inlays demonstrated heightened resistance to accelerated fatigue and reduced susceptibility to cracks in large MOD restorations compared to direct restorations. Although both methods exhibited excellent fatigue performance under physiological masticatory loads, CAD/CAM inlays appear to be particularly suitable for patients with high occlusal forces. In the latter study [36], the authors compared mechanical performance and enamel crack propensity of direct, semi-direct, and CAD/CAM approaches for large MOD composite resin restorations. They found that direct restorations performed as well as inlays when a short fiber-reinforced composite resin base was used.

Özkir [35] and Kim et al. [44] conducted finite element analysis to evaluate stress distribution for different restorations. Özkir concluded that indirect restorations, whether using ceramics or indirect composite resins, had preferable stress distribution and concentration characteristics when compared to direct restorations [35]. In Kim et al.’s study, inlay and onlay ceramic restorations, along with gold crowns covering composite resin filling, were identified as advantageous methods for preventing further crack propagation [44].

Our meta-analysis focused on a specific mechanical property, specifically examining a facet of fracture resistance. This examination involved comparing the behavior of direct and indirect posterior restorations under static compressive forces. Our study showed that there was no difference between direct and indirect restorations for posterior teeth in all cases of restorations with or without cusp coverage and no matter the used direct or indirect material.

The mechanical properties, specifically fracture resistance under compression forces, play a crucial role in the success and longevity of dental restorations. To the knowledge of the authors, this is the first systematic review and comparative analysis of direct and indirect techniques, aiming to provide comprehensive insights into the mechanical properties (static compression forces).

It is challenging to compare direct composite resin and indirect restorations for rehabilitating posterior teeth due to the multitude of factors that could be considered, such as the different cavity configurations, the vast array of available materials on the market, the consideration of whether teeth have undergone endodontic treatment or have undergone an aging procedure (e.g., thermocycling), and even the protocol of adhesion (bonding) employed in each case. Each of these factors can further complicate the comparison and influence the longevity and functionality of the restoration. Finding studies following precisely the same protocol while meticulously addressing all these factors proves challenging. 

Indeed, tooth fracture rate is not affected by factors such as patient’s age, gender, and type and position of tooth (premolar or molar), as demonstrated by Dammaschke et al. [55].

Consequently, this study adopted a focused approach, centering specifically on the type of restorative material employed. Materials were categorized into either composite resin, for both direct and indirect restorations, or ceramic, for indirect restorations. Furthermore, this study meticulously considered whether there was cusp reduction (onlay preparation) or not (inlay preparation), ensuring comparability across groups. Only studies featuring comparable cavity configurations between direct and indirect restorations were included in the quantitative study, thus maintaining consistency, and enabling a more rigorous comparative analysis within the study framework.

Several articles have reported findings that align with the outcomes of this meta-analysis. Notably, the studies of Mergulhão et al. and Yazdi et al., comparing direct composite resin restorations to indirect ceramic restorations, concluded that there is no significant difference in fracture resistance between the two approaches [37,41]. In addition, the studies of Coelho-De-Souza et al., Plotino et al., and Daher et al. found no difference between direct and indirect composite resin restorations [26,27,42].

Other articles have reported divergent findings from those previously mentioned. Camacho et al., Cobankara et al., Ragauska et al., and Bianchi E Silva et al. observed that fracture resistance of indirect ceramic restorations was superior to other restoration types [24,25,28,31].

On the contrary, Bajunaid’s study yielded contrasting results, indicating that direct composite resin restorations exhibited the highest fracture resistance when compared to indirect composite resin and ceramic restorations. Meanwhile, no discernible difference in fracture resistance was found between the indirect restorations [40]. Similarly, Garoushi’s findings indicate that the most effective method for restoring large MOD cavities involves direct restoration using short fiber composite (SFC), either independently or as a bulk core in conjunction with particulate-filled composite (PFC) [46].

Tsertsidou et al.’s study suggests that utilizing CAD/CAM technology and fiber reinforcement techniques in restoring class II MOD cavities offers improvements in terms of fracture resistance compared to resin composite alone. Consequently, indirect composite resin restorations were deemed preferable to direct ones based on this study’s findings [47]. Also, Batalha-Silvaa et al.’s study indicated that indirect composite inlays seem more indicated for high-load patients [30].

Frankenberger et al. compared various preparations and restorations, concluding that less invasive preparation designs did not offer benefits for the stability of post-endodontic restorations, except when using IPS Empress [32].

## 5. Limitations

Our meta-analysis reveals multiple limitations among the reviewed studies. Firstly, the studies showed significant heterogeneity, indicated by a high I^2^. This heterogeneity was exacerbated by the varied materials used in both direct and indirect restorations. Secondly, studies were performed in vitro or using FEA, which may not fully replicate real intra-oral conditions despite attempts to simulate them. Conditions that are different from the real intra-oral conditions, although some attempts were made to simulate them. Thirdly, in this meta-analysis, the evaluation of the mechanical testing was limited to studies relying on static compression fracture resistance testing. Studies employing different mechanical tests such as flexural stresses, cyclic isometric chewing, or evaluating von Mises stress values were not included. It is important to recognize that dental materials often fail due to flexural stresses rather than compressive forces alone [56]. This concern was stated in Ilie et al.’s study, which suggests that while compression tests are frequently reported, they may not be particularly recommended for assessing dental restorative materials like resin composites [57]. Moreover, the area of the restorations was not taken into account, a matter that could be of great importance given the variety of restoration designs. Another limitation is the inclusion of only conventional resin composites for direct restorations, omitting consideration of bulk-fill composites. Similarly, for indirect restorations, only composite resins and ceramics were included, excluding materials like PEEK, as investigated in Prechtel et al.’s study [39]. This selection was made due to the insufficient availability of studies on these materials, thus preventing a quantitative analysis.

## 6. Research Perspective

Expanding the investigation to include a broader range of stressing forces, such as flexural stresses, holds promise for enhancing the clinical relevance of our findings. Exploring the interplay between stress forces, restoration designs, material properties, and environmental factors can provide valuable insights for optimizing treatment outcomes and guiding the development of more durable restorative solutions in the dental practice.

## 7. Conclusions

Based on this study, it can be concluded that the choice between direct and indirect restoration approaches may not significantly impact fracture resistance outcomes in terms of compressive forces. There was no statistically significant difference between direct and indirect restorations for posterior teeth in all cases of restorations with or without cusp coverage and no matter the used material. However, to better evaluate the efficacy of these materials, further studies with more standardized protocols and consideration for oral environment simulation are warranted.

## Figures and Tables

**Table 1:** Search strings.

Database	Search String
Medline	((“mechanical tests” [Mesh] OR “mechanical phenomena” [Mesh] OR “mechanical properties” [tiab] OR “mechanical behavior” [tiab] OR “mechanical performance” [tiab] OR “mechanical strength” [tiab] OR “mechanical evaluation” [tiab] OR “load” [tiab]) AND (“Composite Resins” [Mesh] OR “direct” [tiab]) AND (“indirect” [tiab] OR “Computer-Aided Design” [Mesh] OR “computer-aided design/ manufacturing” OR “Inlays” [Mesh] OR “Onlays” [tiab] OR “Dental Porcelain” [Mesh]) AND (“posterior teeth” [tiab] OR “molar” [Mesh] OR “bicuspid” [Mesh] OR “premolar” [tiab])) Run data search: 3 February 2024 (611 results)
Embase	((mechanical strength OR load) AND (composite resins OR direct) AND (indirect OR computer-aided design OR computer-aided design/manufacturing OR inlays OR onlays OR dental porcelain) AND (posterior teeth OR molar OR bicuspid OR premolar)) Run data search: 3 February 2024 (110 results)
Central	#1‘direct restoration:ti,ab,kw’,#2‘indirect restoration:ti,ab,kw’,#3‘mechanical test:ti,ab,kw’,#4‘#1 AND #2 AND #3‘. Run data search: 3 February 2024 (6 results)

**Table 2:** Characteristics of included studies comparing direct/indirect restorations.

Year of Publication	Author(s)	Study Title	Study Type	Study Population	Endodontic Treatment	Cavity Configuration	Number of Teeth per Group	Evaluated Direct Materials	Evaluated Indirect Materials	Aging Procedure	Mechanical Testing	Evaluation of Fracture Pattern
**2007**	Camacho et al. [24]	Fracture strength of restored premolars	In vitro study	120 maxillar premolars	No	MOD cavities	10	**1. Composite resin (Z-250)** 2. Conventional amalgam restorations (GS-80) 3. Bonded amalgam restorations.	**1. Composite resin (Z-250)** **2. Ceramic (Vitadur Alpha)**	No	Fracture resistance test: static compressive strength	Yes
**2008**	Cobankara et al. [25]	The effect of different restoration techniques on the fracture resistance of endodontically-treated molars	In vitro study	60 mandibular molars	Yes	MOD cavities	10	1. Amalgam **2. Composite resin (Clearfil Photoposterior)** 3. Polyethylene ribbon fiber (Ribbond) + Composite resin	**Ceramic (Estenia)**	Yes	Fracture resistance test: static compressive strength	Yes
**2008**	Coelho-De-Souza et al. [26]	Fracture resistance and gap formation of MOD restorations: influence of restorative technique, bevel preparation and water storage	In vitro study	100 premolars	No	MOD cavities	10	**Composite resin (Filtek Z250)**	**Composite resin (Filtek Z250)**	Yes	Fracture resistance test: static compressive strength	Yes
**2008**	Plotino et al. [27]	Fracture resistance of endodontically treated molars restored with extensive composite resin restorations	In vitro study	45 mandibular molars	Yes	Class II MO cavities + reduction of 2 mesial cusps	15	**Composite resin (Estelite Sigma)**	**Composite resin (Estelite Sigma)**	No	Fracture resistance test: static compressive strength	Yes
**2008**	Ragauska et al. [28]	Influence of ceramic inlays and composite fillings on fracture resistance of premolars in vitro	In vitro study	27 premolars	No	MOD cavities	9	**Composite resin (Filtek P60).**	**Ceramic (Finesse)**	No	Fracture resistance test: static compressive strength	Yes
**2008**	Soares et al. [29]	Influence of restorative technique on the biomechanical behavior of endodontically treated maxillary premolars. Part I: Fracture resistance and fracture mode	In vitro study	70 maxillar premolars	Yes	MOD cavities	10	1. Amalgam **2. Composite resin (Filtek Supreme)**	**1. Composite resin (SR Adoro)** **2. Ceramic (IPS Empress).**	No	Fracture resistance test: static compressive strength	Yes
**2012**	Batalha-Silvaa et al. [30]	Fatigue resistance and crack propensity of large MOD composite resin restorations: Direct versus CAD/CAM inlays	In vitro study	32 maxillar molars	No	MOD cavities	15 for direct 17 for indirect	**Composite resin (Miris2)**	**Composite resin (CEREC inlay with Paradigm MZ100)**	No	Cyclic-load-to-failure test	Yes
**2013**	Bianchi E Silva et al. [31]	Influence of restorative techniques on fracture load of endodontically treated premolars	In vitro study	60 maxillar premolars	Yes	MOD with and without cusp reduction	10	**Four Seasons composite resin (Ivoclar/Vivadent)**	**1. Composite resin (Adoro)** with and **without cusp coverage** **2. Ceramic (IPS Empress)** with and **without cusp coverage**	No	Fracture resistance test: static compressive strength	Yes
**2015**	Frankenberger et al. [32]	Stability of endodontically treated teeth with differently invasive restorations: Adhesive vs. non-adhesive cusp stabilization	In vitro study	264 third molars	Yes	1. MO 2. MOD 3. MO + cusp reduction 4. MOD + cusp reduction	8	1. Bulkfill composite resin (Tetric EvoCeram Bulk Fill) 2. Amalgam	1. Composite resin (IPS Empress) 2. Celtra Duo 3. e.max CAD 4. Lava Ultimate 5. Enamic 6. Gold	Yes	Fracture resistance test: static compressive strength	No
**2016**	Al Amri et al. [33]	Fracture resistance of endodontically treated mandibular first molars with conservative access cavity and different restorative techniques: An in vitro study	In vitro study	72 mandibular first molar teeth	Yes	1. Amalgam cavity 2. Only access cavity 3. Onlay: MOD cavities + cusp reduction 4. Inlay: class I	12	1. Amalgam **2. Composite resin (Tetric_ EvoCeram)**	**1. Ceramic inlay (IPS e.max) with and without cusp coverage** 2. Zirconium crown	No	Fracture resistance test: static compressive strength	Yes
**2016**	Bromberg et al. [34]	Fracture resistance of endodontically treated molars restored with horizontal fiberglass posts or indirect techniques	In vitro study	50 third molars	Yes	MOD cavities (+ cusp reduction for onlays)	10	**1. Composite resin (Filtek Z230 XT)** 2. Transfixed fiberglass post + direct composite resin Filtek Z230 XT (3M ESPE)	**Ceramic (Lava Ultimate)** with and **without cusp coverage**	Yes	Fracture resistance test: static compressive strength	Yes
**2017**	Ozkir [35]	Effect of restoration material on stress distribution on partial crowns: A 3D finite element analysis	FEA	Maxillar first molar tooth	Simulated	MOD + Functional cusps reduction	3	1. Bulkfill composite resin 2. Conventional hybrid composite resin	1. Ceramic 2. Composite resin	-	Von Mises stress values, stress distribution and concentration levels	No
**2017**	Soares et al. [36]	Optimization of large MOD restorations: Composite resin inlays vs. short fiber-reinforced direct restorations	In vitro study	45 maxillar molars	No	MOD cavities	15	Fiber-reinforced composite resin base (EverX Posterior, GC) layered with direct composite (Gra- dia Direct posterior; GC, Lueven, Belgium)	1. Semi-direct inlay (Gradia Direct Posterior; GC, Lueven, Belgium) 2. CAD/CAM inlay (Cerasmart; GC)	Yes	Cyclic-load-to-failure test	Yes
**2019**	Mergulhão et al. [37]	Fracture resistance of endodontically treated maxillary premolars restored with different methods	In vitro study	50 maxillar premolars	Yes	MOD cavities	10	**1. Conventional composite resin (Filtek Z350XT)** 2. Conventional composite resin restoration (Filtek Z350XT) + horizontal glass fiber post (White Post DC) 3. Bulkfill flowable (Filtek) and bulkfill restorative composites (Filtek)	**Ceramic (IPS e-max)**	Yes	Fracture resistance test: static compressive strength	Yes
**2019**	Papadopoulos et al. [38]	Structural integrity evaluation of large MOD restorations fabricated with a bulk-fill and a CAD/CAM resin composite material	In vitro study	51 mandibular molars	No	MOD	17	Bulkfill composite resin (Filtek Bulk-Fill Posterior Restorative)	Composite CAD/CAM inlays (Lava Ultimate)	Yes	Fracture resistance test: static compressive strength	Yes
**2020**	Prechtel et al. [39]	Fracture load of 3D printed PEEK inlays compared with milled ones, direct resin composite fillings, and sound teeth	In vitro study	112 molars	No	Class I + cusp reduction	16	Composite resin (Tetric EvoCeram)	1. Essentium PEEK 2. KetaSpire PEEK MS-NT1 (KET) 3. VESTAKEEP i4 G 4. VICTREX PEEK 450G 5. PEEK JUVORA Dental Disc 2	Yes	Fracture resistance test: static compressive strength	Yes
**2020**	Bajunaid et al. [40]	Influence of type of final restoration on the fracture resistance and fracture mode of endodontically treated premolars with occluso-mesial cavities	In vitro study	60 maxillar premolars	Yes	MO cavities	15	**Composite resin (Filtek Z250)**	**1. Composite resin (Filtek Z250)** **2. Ceramic (IPS E.Max CAD/CAM)**	Yes	Fracture resistance test: static compressive strength	Yes
**2020**	Yazdi et al. [41]	Effect of direct composite and indirect ceramic onlay restorations on fracture resistance of endodontically treated maxillary premolars	In vitro study	45 maxillar premolars	Yes	MOD + cusp reduction	15	**Composite resin (P60)**	**Ceramic (IPS e.max)**	Yes	Fracture resistance test: static compressive strength	Yes
**2021**	Daher et al. [42]	Fracture strength of non-invasively reinforced MOD cavities on endodontically treated teeth	In vitro study	60 mandibular molars	Yes	MOD cavities (+ cusp reduction for onlays)	12	**1. Composite resin (Tetric EvoCeram)** 2. Composite resin + reinforced strip (Tetric EvoCeram + Dentapreg)	**Composite resin (Tetric CAD)** with and **without cusp reduction**	Yes	Fracture resistance test: static compressive strength	Yes
**2021**	Hofsteenge et al. [43]	Influence of preparation design and restorative material on fatigue and fracture strength of restored maxillary premolars	In vitro study	90 maxillar premolars	No	MOD with and without cusp reduction	10	**1. Composite resin (Filtek Supreme XTE) at 3mm with and without cusp reduction** **2. Composite resin (Filtek Supreme XTE) at 5mm with and without cusp reduction**	**1. Ceramic (Shofu Vintage LD Press) at 3mm with and without cusp reduction** **2. Ceramic (Shofu Vintage LD Press) at 5mm with and without cusp reduction**	Yes	Fracture resistance test: static compressive strength	Yes
**2021**	Kim et al. [44]	Occlusal stress distribution and remaining crack propagation of a cracked tooth treated with different materials and designs: 3D finite element analysis	FEA	Mandibular first molar	No	1. Inlay form 2. Onlay form 3. Crown restoration	8	**Composite resin (Filtek Z350)**	**1. Composite resin (Tescera ATL)** **2. Ceramic (Emax)** 3. Gold	-	Von Mises stress values, stress distribution and concentration levels	No
**2023**	Althaqafi [45]	Performance of direct and indirect onlay restorations for structurally compromised teeth	In vitro study	54 mandibular molars	No	MOD cavities + cusp reduction	9	**Composite resin (everX Posterior)**	**1. Composite resin (Grandio)** **2. Ceramic (SHOFU Block HC)**	Yes	Fracture resistance test: static compressive strength	Yes
**2023**	Garoushi et al. [46]	Evaluation of fracture behavior in short fiber-reinforced direct and indirect overlay restorations	In vitro study	120 molars	No	MOD cavities + cusp reduction	15	1. Particulate-filled composite (PFC) (G-aenial Posterior) 2. PFC + different increment of short-fiber composite (SFC) (everX Flow Bulk shade)	1. Cerasmart with SFC 2. Cerasmart without SFC 3. LiSi emax with SFC 4. Lisi emax without SFC	Yes	Fracture resistance test: static compressive strength	Yes
**2023**	Tsertsidou et al. [47]	Fracture resistance of Class II MOD cavities restored by direct and indirect techniques and different materials combination	In vitro study	60 maxillar molars	No	MOD	15	**1. Composite resin (Tetric)** 2. Short-fiber-reinforced composite (EverX posterior Bulk shade) + composite resin 3. Ribbond + composite resin	**Composite resin (Brilliant Crios)**	Yes	Fracture resistance test: static compressive strength	Yes

## Data Availability

The data presented in this study are available in the article.
